# Reframing fruit biofortification for sustainable micronutrient security: from crop innovation to nutritional impact

**DOI:** 10.3389/fpls.2026.1853898

**Published:** 2026-06-08

**Authors:** Tahya Bashir, Rizwana Rashid, Abdul Raouf Malik, A. S. Sundouri, Khalid Mushtaq Bhat, Mohd.Amin Mir, Muneeb Ur Rehman, Mariyam Masood, Tehleelah

**Affiliations:** 1 Division of Fruit Science, Faculty of Horticulture, Sher-e-Kashmir University of Agricultural Sciences & Technology of Kashmir, Srinagar, India; 2 Division of Food Science and Technology, Faculty of Horticulture, Sher-e-Kashmir University of Agricultural Sciences & Technology of Kashmir, Srinagar, India

**Keywords:** bioavailability, fruit biofortification, genome editing, hidden hunger, micronutrient malnutrition, nutritional security, sustainable food systems

## Abstract

Micronutrient malnutrition, commonly referred to as “hidden hunger, “ remains a persistent global health challenge, particularly in regions with limited dietary diversity. Although agricultural intensification has substantially improved caloric availability, it has not ensured adequate micronutrient density in food systems, highlighting the urgent need for nutrition-sensitive crop improvement strategies. Biofortification has emerged as a sustainable and cost-effective approach to enhance the micronutrient content of food crops through agronomic, genetic, and biotechnological interventions. While biofortification research has predominantly focused on staple cereals and legumes, horticultural fruit crops have received comparatively limited attention despite their widespread consumption, high consumer acceptance, and natural richness in bioactive compounds. This review advances beyond existing overviews by providing a critical and comparative evaluation of agronomic, conventional breeding, and biotechnological approaches for fruit crop biofortification, with particular emphasis on their effectiveness, scalability, limitations, and translational potential. Current advances aimed at enhancing iron, zinc, iodine, selenium, and provitamin A concentrations are comprehensively synthesized. Special attention is given to the physiological and molecular mechanisms regulating micronutrient uptake, transport, accumulation, and storage in fruit tissues. In addition, advanced biotechnological tools, including CRISPR/Cas-mediated genome editing, are critically assessed in relation to biosafety, regulatory considerations, and practical applicability. Evidence from major fruit crops, including apple, banana, mango, pomegranate, strawberry, and papaya, demonstrates that integrated biofortification strategies can improve micronutrient density while maintaining fruit yield and quality. Importantly, this review addresses a major knowledge gap by linking crop-level nutrient enhancement with micronutrient bioavailability and human nutritional outcomes, emphasizing the influence of food matrix interactions and nutrient absorption efficiency. Key constraints, including genotype × environment interactions, postharvest nutrient instability, climate-driven variability, and limited clinical validation, are also discussed. Finally, a systems-level framework integrating plant science, human nutrition, postharvest biology, and policy perspectives is proposed to support the large-scale adoption of nutrition-sensitive fruit biofortification. Collectively, fruit crop biofortification represents a promising strategy for improving global micronutrient security and advancing sustainable food systems

## Graphical Abstract

**Figure:**
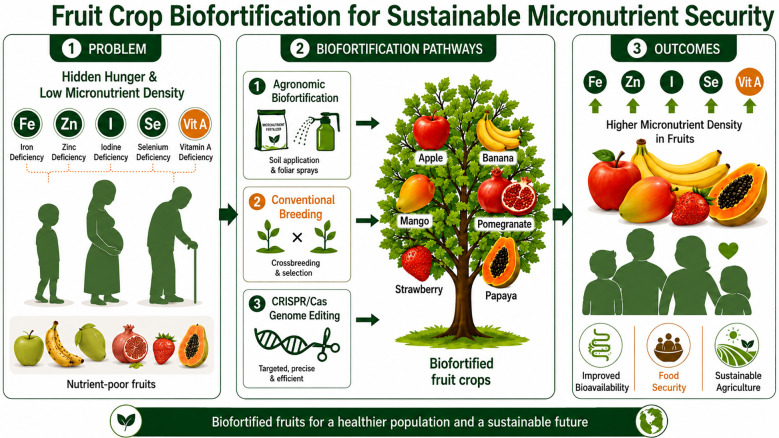

## 1 Introduction

Agriculture remains fundamental to global food security, sustaining billions of people and serving as a cornerstone of efforts to combat hunger and malnutrition. Over the past several decades, advances in agricultural technologies, crop genetics, and production systems have substantially increased food availability, enabling the caloric demands of a rapidly growing global population to be met. However, the productivity-driven paradigm established during the Green Revolution largely prioritized yield maximization over nutritional quality, often contributing to declines in micronutrient density and dietary diversity. Consequently, contemporary food security discourse has shifted beyond caloric sufficiency toward broader concepts of nutritional security and diet quality, recognizing that adequate energy intake alone is insufficient to ensure optimal human health.

Micronutrient malnutrition, commonly referred to as “hidden hunger, “ represents one of the most pervasive forms of global malnutrition, characterized by adequate caloric intake but insufficient consumption of essential vitamins and minerals (Stevens et al., 2022). Recent estimates indicate that billions of individuals worldwide fail to meet recommended dietary intakes of critical micronutrients, with more than five billion people experiencing inadequate intake of iodine (68%), vitamin E (67%), and calcium (66%), and over four billion exhibiting insufficient intake of iron (65%), riboflavin (55%), folate (54%), and vitamin C (53%) (Passarelli et al., 2024). The burden of these deficiencies is not uniformly distributed. Women are disproportionately affected by deficiencies in iodine, vitamin B12, iron, and selenium, whereas men exhibit comparatively higher inadequacies of magnesium, vitamin B6, and zinc. Among all micronutrient deficiencies, those involving iron, zinc, and vitamin A remain the most clinically significant, particularly among women and children in low- and middle-income countries. Vitamin A deficiency (VAD), in particular, continues to represent a major public health challenge, contributing to more than 600, 000 deaths annually, especially among children under five years of age (WHO, 2021; Stevens et al., 2015).

In India, micronutrient malnutrition remains a persistent and multifaceted public health concern. According to the State of Food Security and Nutrition in the World (SOFI) 2025 report, 21.9% of the population continues to live in extreme poverty, accompanied by high prevalence of undernutrition, childhood stunting (34.8% among children under five years), and anemia (58.4%), much of which is associated with iron deficiency (Unicef, 2025; Yadava et al., 2020). These trends highlight the urgent need for sustainable, food-based strategies capable of improving micronutrient intake at the population level while complementing existing supplementation and fortification programs.

**Figure 1:**
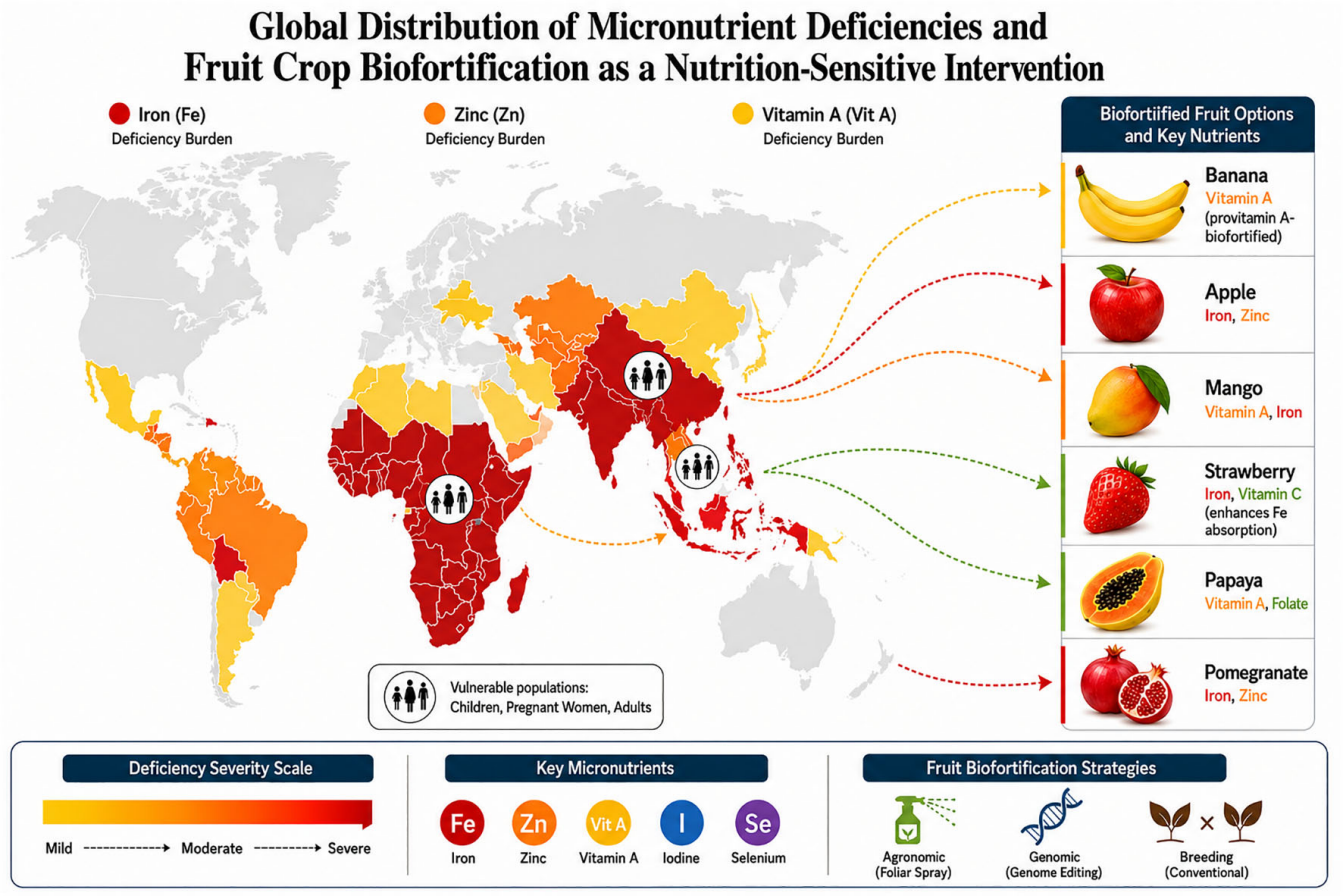
Global distribution of iron, zinc, and vitamin A deficiencies and the potential of fruit crop biofortification as a nutrition-sensitive intervention. Regions with high micronutrient deficiency are highlighted, with biofortified fruits presented as targeted dietary solutions for improved nutritional security.

Within this context, biofortification has emerged as a promising and economically sustainable strategy for enhancing the micronutrient content of food crops through agronomic interventions, conventional breeding, and advanced biotechnological approaches. To date, however, the majority of biofortification research and implementation efforts have focused predominantly on staple cereals and legumes. In contrast, fruit crops despite their widespread consumption, high consumer acceptance, and intrinsic nutritional value have received comparatively limited critical attention. This represents a significant gap, as fruits are naturally rich in vitamins, sugars, organic acids, antioxidants, and diverse bioactive compounds that contribute substantially to human health and may enhance micronutrient bioavailability (Walia et al., 2022). Moreover, in many micronutrient-deficient regions, fruits constitute an important component of daily diets, thereby providing a strategically relevant platform for nutrition-sensitive interventions (**Figure 1**).

Among horticultural crops, banana and mango are particularly noteworthy because of their dietary importance and extensive cultivation across regions affected by hidden hunger. Biofortified bananas enriched with provitamin A, iron, and zinc have been proposed as potential public health interventions in countries such as Uganda, whereas nutrient-enhanced mangoes are being explored in India, the United States, and Dominica (Jha et al., 2025). Unlike supplementation or pharmaceutical interventions, fruit biofortification offers the advantage of integrating improved nutrition directly into habitual dietary patterns, thereby increasing the likelihood of long-term adoption, scalability, and sustainability.

The term “reframing” in the title of this review reflects a deliberate conceptual shift in how fruit crop biofortification is interpreted and evaluated. Existing reviews have largely treated biofortification as a nutrient-enrichment strategy focused primarily on increasing micronutrient concentrations in edible tissues, often emphasizing staple crops and providing largely descriptive overviews of available technologies. In contrast, this review positions fruit biofortification within a broader systems-oriented framework that integrates crop improvement with plant physiological mechanisms, nutrient bioavailability, postharvest stability, and translational nutritional outcomes. Importantly, comparatively limited attention has been devoted to the molecular and physiological processes governing micronutrient uptake, transport, sequestration, remobilization, and accumulation in fruit tissues, despite the fact that these processes fundamentally determine the nutritional efficacy of biofortification strategies.

Accordingly, this review advances beyond conventional narrative summaries by critically comparing agronomic, conventional breeding, and biotechnological approaches in terms of their effectiveness, scalability, sustainability, biosafety, and translational potential specifically within fruit crops. In addition, this review critically evaluates key limitations currently constraining the field, including genotype × environment interactions, climate-driven nutrient variability, postharvest nutrient degradation, regulatory and socio-economic barriers, and the limited availability of robust clinical evidence linking crop-level nutrient enhancement with measurable improvements in human nutritional status.

Collectively, these considerations position fruit crop biofortification as a critical component of nutrition-sensitive agriculture with substantial potential to contribute to micronutrient security, public health resilience, and sustainable food systems. By integrating advances in plant science, human nutrition, biotechnology, and policy frameworks, fruit biofortification may provide a viable pathway toward addressing persistent micronutrient deficiencies in an increasingly food-insecure and climate-vulnerable world.

## 2 Micronutrients and the impact of their deficiencies on health

Micronutrients, encompassing essential minerals and vitamins, are required in small quantities but play fundamental roles in maintaining physiological homeostasis, growth, metabolism, immune function, and overall health. Despite their relatively low dietary requirements, inadequate intake imposes a substantial global health burden. South Asia, in particular, continues to experience a disproportionately high prevalence of micronutrient malnutrition (Harding et al., 2018), with iron, zinc, and vitamin A deficiencies remaining among the most widespread worldwide (Passarelli et al., 2024). Importantly, both deficiency and excess of micronutrients can disrupt physiological balance, underscoring the necessity for optimal intake levels (Espinosa-Salas and Gonzalez-Arias, 2023).

At the molecular level, micronutrients are indispensable for numerous biological processes, including gene regulation, enzymatic catalysis, hormone synthesis, and cellular protection against oxidative stress (Cena and Calder, 2020; Shenkin, 2006). Consequently, their deficiencies manifest in a wide spectrum of health disorders, ranging from impaired cognitive development and weakened immune responses to metabolic dysfunction and increased susceptibility to disease. These challenges highlight the urgency of adopting nutrition-sensitive approaches to improve micronutrient intake at the population level.

From a functional and nutritional perspective, essential micronutrients are broadly categorized into micro-minerals and vitamins. A summary of key micronutrients, their biological functions, deficiency impacts, and relevance to biofortification strategies is presented in **Table 1**. Although fruits contribute significantly to dietary intake of several vitamins and selected minerals, their concentrations are often insufficient to meet recommended dietary allowances (RDAs), particularly in populations with limited dietary diversity. This gap underscores the importance of biofortification as a sustainable strategy to enhance the nutritional quality of fruit crops and improve micronutrient intake.

**Table 1:** Key micronutrients, their biological roles, deficiency impacts, and relevance to biofortification strategies.

Nutrient	Biological function	Deficiency impact	Biofortification priority	Key references
Iron (Fe)	Oxygen transport (haemoglobin), electron transfer, enzymatic activity	Anemia, impaired cognitive development, reduced immunity, fatigue	Very high – most widespread deficiency globally; strong clinical and biofortification evidence	Bouis and Saltzman (2017); Abbaspour et al. (2014); White and Broadley (2009)
Zinc (Zn)	Enzyme activation, gene expression, immune regulation, DNA synthesis	Growth retardation, immune dysfunction, increased infection susceptibility	Very high – critical for child health; major target in global biofortification programs	Bouis and Saltzman (2017); Prasad (2013); White and Broadley (2009)
Provitamin A carotenoids	Vision, immune function, epithelial integrity	Night blindness, xerophthalmia, increased child mortality	Very high – global public health priority; successful biofortification interventions	Saltzman et al. (2013); West (2002); Bouis and Saltzman (2017)
Iodine (I)	Thyroid hormone synthesis, metabolic regulation, neurodevelopment	Goitre, hypothyroidism, impaired cognitive development	High – limited plant availability; requires agronomic interventions	White and Broadley (2009); Zimmermann and Boelaert (2015)
Selenium (Se)	Antioxidant defence, selenoprotein function, immune support	Cardiomyopathy, impaired immunity, oxidative stress-related disorders, thyroid disorders, autoimmune and neurodegenerative diseases	Moderate–high – region-specific deficiency; responsive to soil/foliar enrichment	White and Broadley (2009); Rayman (2012); Ekumah et al., 2021; Duţă et al., 2025.
Magnesium (Mg)	Enzyme cofactor, ATP metabolism, neuromuscular function	Muscle weakness, metabolic imbalance	Moderate – supportive role in metabolic health	White and Broadley (2009)
Vitamin C (Ascorbic acid)	Antioxidant activity, enhances iron absorption	Scurvy, reduced iron bioavailability	Indirect – improves effectiveness of iron biofortification	White and Broadley (2009); Mayer et al. (2008)

### 2.1 Iron deficiency

Iron deficiency, also referred to as sideropenia, occurs when body iron stores are insufficient to sustain essential physiological processes. It is the most prevalent micronutrient deficiency globally and the leading cause of nutritional anemia, affecting an estimated two billion people worldwide (Hanif and Anwer, 2023; Abbaspour et al., 2014). Iron plays a central role in oxygen transport, electron transfer, and enzymatic reactions; therefore, its deficiency extends beyond hematological disorders. It is strongly associated with impaired cognitive development, reduced physical work capacity, compromised immune function, and increased susceptibility to infections. Women of reproductive age, infants, and young children are particularly vulnerable due to increased physiological demands and limited dietary intake. Given its widespread prevalence and severe health implications, iron has been a primary target of global biofortification initiatives, with substantial evidence supporting its effectiveness in improving human nutritional status (Bouis and Saltzman, 2017; White and Broadley, 2009).

### 2.2 Zinc deficiency

Zinc deficiency represents another major global health challenge, particularly in low- and middle-income countries where diets are often cereal-based and low in bioavailable zinc. Zinc is an essential trace element involved in numerous biological processes, including enzyme activation, gene expression, cellular signaling, DNA synthesis, and antioxidant defense (Chu et al., 2025; Prasad, 2013).

Inadequate zinc intake is associated with increased susceptibility to infections, impaired immune responses, delayed wound healing, growth retardation in children, skin disorders, and diarrheal diseases (Stiles et al., 2024). Due to its critical role in immune modulation and child development, zinc deficiency significantly contributes to global morbidity and mortality. Consequently, zinc biofortification has emerged as a key strategy in crop improvement programs aimed at enhancing dietary zinc intake in vulnerable populations (Bouis and Saltzman, 2017; White and Broadley, 2009).

### 2.3 Iodine deficiency

Iodine is an essential micronutrient required for the synthesis of thyroid hormones, thyroxine (T4) and triiodothyronine (T3) which regulate metabolic activity, growth, and neurodevelopment. Inadequate iodine intake disrupts thyroid function and can result in a range of disorders, including goitre, hypothyroidism, fatigue, and impaired cognitive performance (Hatch-McChesney and Lieberman, 2022; Zimmermann and Boelaert, 2015). Iodine deficiency is particularly detrimental during pregnancy, as it can impair fetal brain development and lead to irreversible intellectual disabilities. Although iodized salt programs have significantly reduced its prevalence, iodine deficiency persists in certain regions. From a biofortification perspective, iodine enrichment of crops through agronomic approaches has gained increasing attention as a complementary strategy to improve dietary iodine intake (White and Broadley, 2009).

### 2.4 Vitamin A (retinol) deficiency

Vitamin A is a fat-soluble micronutrient essential for vision, immune competence, epithelial integrity, and embryonic development. It also plays a crucial role in antioxidant defense and cellular differentiation (Günes-Bayir, 2025). Vitamin A deficiency remains a major public health concern and is one of the leading causes of preventable blindness in children. Clinical manifestations include night blindness, xerophthalmia, and, in severe cases, keratomalacia (Diab and Krebs, 2018; West, 2002).

In addition to ocular complications, inadequate vitamin A status compromises immune function, increasing susceptibility to infectious diseases and mortality risk among children. Given its global significance, provitamin A biofortification has been widely implemented in staple crops and is increasingly being explored in fruit crops such as banana and mango to enhance dietary intake (Saltzman et al., 2013; Bouis and Saltzman, 2017).

### 2.5 Vitamin B complex deficiencies

The vitamin B complex comprises a group of water-soluble vitamins that play essential roles in energy metabolism, neurological function, and hematopoiesis. Thiamine (vitamin B1) is critical for carbohydrate metabolism, and its deficiency may result in beriberi and Wernicke–Korsakoff syndrome. Pyridoxine (vitamin B6) is involved in amino acid metabolism and neurotransmitter synthesis; its deficiency can lead to anemia, neurological disturbances, and mood disorders. Folate (vitamin B9) is indispensable for DNA synthesis and cell division, and its deficiency is associated with megaloblastic anemia and an increased risk of neural tube defects during pregnancy. Vitamin B12 (cobalamin) deficiency can cause pernicious anemia, neuropathy, and cognitive impairment (Sarwar and Sarwar, 2022). Collectively, deficiencies in B vitamins can severely impair metabolic and neurological health.

## 3 Biofortification strategies

Biofortification encompasses a suite of approaches aimed at enhancing the micronutrient density of crops through genetic, agronomic, and biotechnological interventions. These strategies differ in their underlying mechanisms, scalability, cost-effectiveness, and long-term sustainability. The principal biofortification approaches are illustrated in **Figure 1**.

### 3.1 Agronomic biofortification

Agronomic biofortification represents one of the most immediately applicable strategies for enhancing micronutrient concentrations in crops through the targeted application of micronutrient-enriched fertilizers. By increasing the availability of essential mineral elements within the soil–plant system, this approach seeks to enhance nutrient uptake, translocation, and accumulation in edible tissues. Applications in agronomic biofortification are generally delivered through either soil incorporation or foliar spraying, both of which aim to enhance micronutrient acquisition and accumulation in edible plant tissues. However, the physiological pathways underlying these approaches differ substantially and strongly influence biofortification efficiency. In foliar applications, micronutrients penetrate the leaf surface through the cuticle and stomata and subsequently enter the phloem, thereby bypassing the comparatively slower and often less efficient root uptake pathway. This direct route frequently results in more rapid nutrient translocation and improved delivery to developing sink tissues, making foliar application a particularly effective strategy for fruit biofortification (Blindauer and Schmid, 2010). Consistent with this, foliar application of selenate has been reported to produce higher selenium accumulation in fruits compared with soil-applied selenate, primarily due to enhanced retention and redistribution through leaf tissues (Lin et al., 2024).

**Figure 2:**
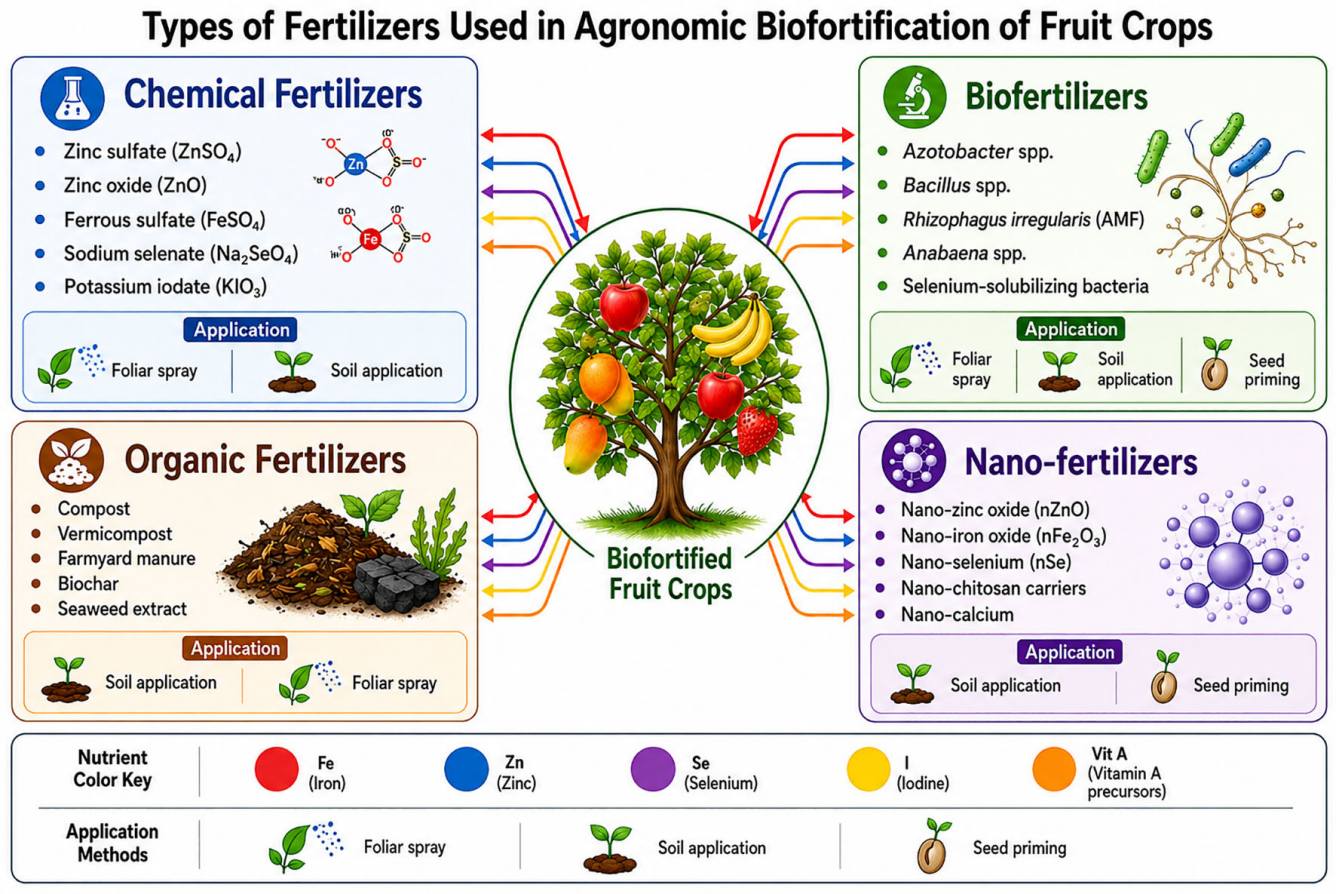
Types of fertilizers and application methods used in agronomic biofortification, including soil and foliar applications.

Agronomic biofortification is especially effective for mineral nutrients, whereas vitamins are generally synthesized through endogenous metabolic pathways and therefore exhibit comparatively limited responsiveness to direct fertilizer-based interventions (Çakmak and Kutman, 2018). The major fertilizer types and application methods employed in agronomic biofortification are illustrated in **Figure 2**. Nevertheless, the efficiency of micronutrient delivery is strongly dependent on the method, timing, and formulation of nutrient application. Soil application primarily improves nutrient availability within the rhizosphere and supports sustained root uptake, whereas foliar spraying enables rapid and targeted nutrient absorption during critical developmental stages. Recent field studies further demonstrate the broader nutritional implications of these approaches. For example, foliar application of sodium selenate at 150 g ha^−^¹ in raspberry, blueberry, red currant, honeysuckle, and apple orchards significantly increased selenium concentrations in fruit-derived juices while simultaneously improving nutritionally relevant parameters, including antioxidant activity, ferulic acid, and resveratrol content (Mezey et al., 2025). Such findings suggest that agronomic biofortification may influence not only micronutrient concentration but also the broader phytochemical and functional quality profile of fruits.

Nevertheless, agronomic biofortification has demonstrated considerable promise in horticultural systems. Studies in fruit crops including apple, banana, and mango have reported significant improvements in mineral concentration, antioxidant activity, and overall fruit quality following targeted micronutrient applications, as summarized in **Table 2**. Collectively, these findings highlight the practical applicability of agronomic interventions while emphasizing the need to optimize fertilizer formulation, dosage, application timing, and delivery method to maximize nutritional outcomes.

**Table 2:** Agronomic biofortification of various crops.

S. No.	Fruit crop	Micronutrient treatment (application method & dose)	Country/region	Reference
1	Apple	Foliar application of iodine (0.5 kg ha^−^¹), selenium (0.25 kg ha^−^¹), and calcium (7 kg ha^−^¹)	Poland	(Wójcik, 2023)
2	Banana	Pseudostem injection of ZnSO_4_ solutions (1–4%)	Brazil	(Guevara et al., 2021)
3	Pomegranate	Foliar spray of zinc and boron (0.3–0.6%)	India	(Guleria et al., 2025)
4	Mango	Foliar application of nano-Zn (100 ppm), ZnSO_4_ (0.1%), and chelated Zn (0.2%)	Saudi Arabia	(Makhasha et al., 2024)
5	Strawberry	Selenium application as Na_2_SeO_4_ (10–100 µM, nutrient solution/foliar)	Italy	(Mimmo et al., 2017)
6	Grape	Foliar Zinc application as ZnSO_4_ (0.2-0.5%) during berry development	Iran	(Tariq et al., 2020)
7	Papaya	Foliar application of micronutrient mixture of Iron, Boron and Zinc	India	(Prakasha and Preethi, 2025)
8	Citrus (*Citrus aurantifolia* Swingle)	Foliar application micronutrinets Zn (0.1%), Cu (0.05%) and B (0.05%)	Nepal	(Bastakoti et al., 2022)
9	Guava	Soil and foliar application of ZnSO_4_ (0.4%) and Borax (0.2%)	India	Adak et al., 2023
10	Pear	Foliar application of 1.5% ZnEDTA	China	Liu et al., 2023
11	Grapes	Foliar application of ZnO and ZnSO_4_ (150, 450 and 900 g ha^-1^)	Portugal	Daccak et al., 2022
12	Mango	Foliar apllication of ZnSO_4_ and Fe SO_4_ (0.05%)	Inida	Mahida et al., 2023

Importantly, increasing evidence indicates that integrated nutrient management strategies combining soil and foliar applications often achieve greater micronutrient accumulation than either approach alone, particularly when nutrients are supplied during physiologically critical growth stages such as flowering, fruit set, and fruit development (Jangra et al., 2024). These observations underscore the importance of synchronizing nutrient delivery with plant developmental dynamics to maximize nutrient uptake efficiency and partitioning into edible tissues.

However, the effectiveness of agronomic biofortification extends far beyond external nutrient supply alone. The ultimate accumulation of micronutrients in fruits is governed by a highly coordinated network of physiological and molecular processes, including root acquisition from the rhizosphere, membrane transport, xylem and phloem loading, intracellular sequestration, long-distance redistribution, and remobilization toward developing sink tissues.

Importantly, these processes are strongly influenced by genotype, soil physicochemical properties, environmental conditions, and crop management practices, resulting in substantial variation in nutrient use efficiency and micronutrient accumulation across fruit crops and production systems. Consequently, successful biofortification depends not only on fertilizer application but also on the plant’s intrinsic capacity to acquire, transport, partition, and retain micronutrients within edible organs. Despite their central importance, these mechanistic aspects have received comparatively limited attention in fruit biofortification research, where emphasis has often remained focused on agronomic outcomes rather than the underlying biological processes controlling nutrient accumulation.

A mechanistic understanding of micronutrient uptake, transport, and partitioning is therefore essential for improving nutrient use efficiency, optimizing biofortification strategies, and achieving stable nutritional enhancement under diverse agro-environmental conditions. The following section summarizes the principal physiological and molecular mechanisms governing micronutrient acquisition and accumulation in fruit crops.

#### 3.1.1 Physiological and molecular processes in nutrient transport and biofortification

Micronutrients are required in lesser quantities by plants than macronutrients for their growth and development (Hansch and Mendel, 2009). Plant micronutrients include iron (Fe), manganese (Mn), copper (Cu), zinc (Zn), boron (B), molybdenum (Mo), chloride (Cl) and nickel (Ni). The availability of these micronutrients varies greatly depending on the soil conditions. For example, fall in the soil pH by one unit, increases the Zn concentration in the soil solution by 100 times (Rengel, 2015). The initial stage of nutrient acquisition and accumulation in plants is via roots which is followed by xylem loading. Therefore, understanding the forms of micronutrients present in the soil and how they are absorbed by plants is crucial to consider for enhancing plant nutrition (Bhardwaj et al., 2022). Plants have developed highly specialized and tightly regulated mechanisms for nutrient acquisition. Among them, transporters play a pivotal role in maintaining ion homeostasis by mediating the selective movements of ions across the membrane (Vishwakarma et al., 2019; Huang et al., 2020; Ma and Tsay, 2021). In dicots and non-graminaceous species, Fe acquisition relies on mechanisms adapted to environments with limited Fe availability. Under such conditions, an active proton pump increases the solubility of Fe+3 via ferric chelate reductase which reduces the Fe +3 to the more soluble ferrous form (Fe+2). Then, Fe +2 is transported into root cells via Fe +2 transporters. In contrast, graminaceous monocot’s root secrete phytosiderophores that chelate Fe+3 in the soil and takes up this stable complex via specific transporters (Hell and Stephan, 2003). Fe deficiency limits both the fruit quality and overall yield in fruit trees, particularly in pear trees growing in calcareous soils (Donnini et al., 2011). In genus *Malus*, a mutant allel of IRT1 has been identified that enhances the IRT1, which allows apple plant to adapt to Fe deficiency (Zhang et al., 2017). In apple, 18 Fe regulated transporter like protein (ZIP) family genes have been identified. In *Arabidopsis thaliana*, a transgenic plant, an iron transporter MdZIP10 not only rescues the growth of Fe2+ uptake-defective yeast mutants but also enhances Fe accumulation and alleviates the deficiency symptoms of Fe (Ma et al., 2019). Some transporters facilitate the uptake of key nutrients like zinc (Zn) (Assunção et al., 2010) and copper (Cu). IRT1 is also responsible for the uptake of elements like calcium (Ca) (Connolly et al., 2002), manganese (Mn), cd and zinc (Zn) (Cohen et al., 1998; Vert et al., 2002). Zn deficiency induces IRT3 and ZIP4 genes which are involved in increasing Zn in xylem (Grotz et al., 1998). Genes such as ZIP2, ZIP4, ZIP5 and ZIP9, expressed in roots and leaves are up regulated during Zn deficient conditions and gene ZIP2 and ZIP4 are transcriptionally regulated by copper (Cu) (Wintz et al., 2003). In case of Se, it was initially believed to enter plant roots by diffusion, but later studies confirmed the active root uptake of selenium by low and high affinity transporters (Shrift and Ulrich, 1969; Zhang et al., 2003; Li et al., 2008). Plant roots employ different transport pathways for selenate (SeO_4_^2-^) and selenite (SeO_3_^2-^), which differs between selenium non-accumulator and hyperaccumulator species (Lima et al., 2018). SULTR1 transporters are high affinity S- transporters present in root cells (Sors et al., 2005; Shinmachi et al., 2010). Since plants lack dedicated selenium transporters, selenium uptake predominantly occurs via the sulfar transport pathways. Evidence indicated that the root absorption is mediated by SULTR1, followed by xylem loading through SULTR2 and transport into chloroplasts via SULTR3, where selenate will be reduced to selenite and incorporated into organic compounds (Wang et al., 2025).

When micronutrients enter xylem, leaves act as a sink for both nutrients and carbohydrates. The nutrients are transported upwards by transportational pull and deposited in the leaves. As the plant continues to grow, leaves shift from being the sink to sources. The nutrient movement from roots to leaves occurs through xylem while redistribution from leaves to other plant organs like fruits or grains take place via the phloem. The elements like selenium (Se) and magnesium (Mg) exhibit high phloem mobility while elements like Fe, Cu, Ca and iodine (I) show restricted movement. However, the processes of xylem unloading and phloem loading remains poorly understood with limited insights into nutrient translocation to edible plant parts (White and Broadley, 2003). Therefore, biofortification strategies aimed at enhancing micronutrient density must consider not only nutrient supply but also plant physiological efficiency and nutrient partitioning. The success of these strategies ultimately depends on the plant’s capacity to acquire, transport, partition and retain micronutrients within edible tissues.

Despite its operational simplicity and relatively rapid effectiveness, agronomic biofortification remains constrained by several agronomic, economic, and environmental limitations. A major limitation is that nutrient enhancement achieved through fertilizer application is inherently non-heritable and therefore requires repeated external inputs across production cycles (White and Broadley, 2009; Cakmak et al., 2010). In addition, the large-scale adoption of agronomic biofortification may be restricted by the recurring costs associated with micronutrient-enriched fertilizers and by environmental concerns related to repeated fertilizer application (Dhotra et al., 2021). The use efficiency of several key micronutrients, particularly iron, zinc, and copper, also remains relatively low, often ranging between 1–5%, due to soil fixation, leaching losses, and complex interactions with soil physicochemical properties that restrict plant uptake and translocation to edible tissues (Alshaal and El-Ramady, 2017; Bhardwaj et al., 2022). Furthermore, excessive or prolonged micronutrient application may disrupt nutrient homeostasis, contribute to residual soil accumulation, and increase the risk of phytotoxicity and environmental contamination (Jha et al., 2025).

Consequently, agronomic biofortification alone is unlikely to provide a fully sustainable long-term solution to micronutrient malnutrition. Rather, its greatest potential may lie in integration with complementary strategies such as conventional breeding and genetic engineering to achieve more durable, scalable, and nutritionally stable outcomes. Such integrated approaches can enhance the overall effectiveness of biofortification programs while simultaneously addressing the biological and environmental limitations associated with individual strategies (Singh et al., 2025).

Critically, however, the current literature also reveals important inconsistencies and unresolved challenges that warrant closer examination. While many studies report substantial increases in micronutrient concentrations at harvest, comparatively few evaluate the stability of these nutritional gains during postharvest storage, transportation, and commercial distribution. This represents a significant limitation because micronutrient retention may decline substantially depending on storage duration, temperature, processing conditions, and fruit physiology. For example, agronomic biofortification of melon with iodine salts (KI and KIO_3_) increased fruit iodine concentration at harvest, yet subsequent storage at 25 °C for 5–15 days resulted in measurable declines in both nutrient retention and fruit quality parameters (Wójcik, 2023; Groth et al., 2020; Soriano-Melgar et al., 2025). These observations raise an important translational concern: studies focusing exclusively on nutrient concentration at harvest may overestimate the nutritional benefits reaching consumers. Therefore, future research should increasingly incorporate postharvest nutrient stability, bioavailability, and real-world supply chain dynamics into agronomic biofortification assessments to evaluate their nutritional efficacy and public health relevance more accurately.

### 3.2 Conventional breeding

Conventional breeding remains a cornerstone strategy in crop biofortification, enabling the development of nutrient-dense cultivars through systematic selection, hybridization, and recombination. By exploiting naturally occurring genetic variability within and across germplasm pools, this approach facilitates the enhancement of mineral and vitamin concentrations in edible plant tissues. A key advantage of conventional breeding lies in its long-term sustainability, as improved nutritional traits are genetically inherited and stably expressed across successive generations (Saltzman et al., 2017). Consequently, once biofortified cultivars are developed and disseminated, they provide a cost-effective and durable solution for mitigating micronutrient deficiencies.

The success of this approach is largely dependent on the availability of sufficient genetic variation for target nutrients within the crop gene pool. In cases where such variability is limited, desirable traits may be introgressed from wild relatives or genetically diverse lines through pre-breeding and introgression breeding strategies, followed by recurrent selection to incorporate these traits into agronomically superior cultivars (Hirschi, 2009). Although this process is time-intensive, it ensures stable integration of enhanced nutritional traits without compromising the genetic integrity and adaptability of the crop.

In addition to conventional hybridization, mutation breeding has been employed to broaden the genetic base for biofortification. This approach involves the induction of heritable genetic variation using physical mutagens (e.g., gamma irradiation) or chemical agents, followed by selection of superior nutrient-enriched lines (Sheoran et al., 2022). Mutation breeding has contributed to the development of improved fruit cultivars with enhanced nutritional profiles.

A notable example is the pomegranate cultivar ‘Solapur Lal’, developed by ICAR–National Research Centre on Pomegranate, Pune, India. This variety exhibits significantly enhanced micronutrient content, along with high yield potential (23–27 t ha^−^¹) and adaptability to semi-arid agro-climatic conditions (Yadava et al., 2020). Comparative nutritional profiling of ‘Solapur Lal’ and the widely cultivated ‘Ganesh’ cultivar is presented in **Table 3**. Such case studies underscore the practical relevance of conventional breeding approaches in horticultural biofortification, particularly where trait stability, consumer acceptance, and regional adaptability are critical.

**Table 3:** Comparative nutrient composition of pomegranate cultivars ‘Ganesh’ and ‘Solapur Lal’.

Nutrient	Ganesh	Solapur Lal	Unit	Reference
Iron (Fe)	2.7–3.2	5.6–6.1	mg/100 g	(Yadava et al., 2020)
Zinc (Zn)	0.50–0.54	0.64–0.69	mg/100 g	
Vitamin C	14.2–14.6	19.4–19.8	mg/100 g	

### 3.3 Genetic engineering

Genetic engineering represents a biotechnology-driven approach to biofortification, enabling precise and targeted modification of plant genomes to enhance nutritional traits. This strategy includes the introduction of exogenous genes, overexpression of endogenous genes, gene silencing through RNA interference (RNAi), and targeted genome modification using advanced genome-editing tools such as CRISPR/Cas systems (Kiran, 2020). Unlike conventional breeding, genetic engineering is not restricted by species barriers, thereby significantly expanding the range of traits that can be incorporated into crop plants.

Through targeted manipulation of metabolic pathways, genetic engineering facilitates enhanced biosynthesis, accumulation, and distribution of micronutrients in edible plant tissues. Biofortification strategies using this approach primarily focus on genes involved in nutrient biosynthesis, transport, sequestration, and bioavailability. In addition, efforts have been made to reduce antinutritional compounds that inhibit nutrient absorption, thereby improving overall nutritional efficiency (Arjoo and Shreya, 2024).

A landmark example of successful genetic biofortification is *Golden Rice*, which was engineered to accumulate β-carotene (provitamin A) in the endosperm to combat vitamin A deficiency (VAD) (Prasad et al., 2015). By introducing key genes involved in the carotenoid biosynthetic pathway, this innovation demonstrated the feasibility of metabolic engineering for improving nutritional quality in staple crops.

Similar approaches have been extended to fruit crops. For instance, provitamin A enriched “Golden Bananas, “ developed under the leadership of Professor James Dale at Queensland University of Technology, were engineered to accumulate elevated levels of β-carotene in the fruit pulp. This initiative, supported by the Bill & Melinda Gates Foundation, targeted populations in Uganda where bananas serve as a dietary staple (Dale et al., 2017). The enhanced carotenoid accumulation results in a characteristic golden-orange pulp and represents a significant advancement in fruit crop biofortification (Quiroz-Iturra et al., 2017).

Despite its high precision and effectiveness, genetic engineering raises several ecological, regulatory, and societal concerns. One major environmental issue is gene flow, defined as the unintended transfer of transgenes to wild relatives or non-target species, which may have ecological implications (Winkler, 2011). Furthermore, public perception and regulatory frameworks continue to influence the adoption of genetically modified crops in many regions.

To address these challenges, genome-editing technologies have emerged as next-generation tools for crop improvement. Programmable nucleases such as meganucleases, zinc-finger nucleases (ZFNs), transcription activator-like effector nucleases (TALENs), and CRISPR/Cas systems enable precise insertion, deletion, or modification of DNA sequences. Among these, CRISPR/Cas9 has gained prominence due to its simplicity, high efficiency, and improved target specificity compared to earlier platforms (Chen and Gao, 2013; Gaikwad et al., 2025). Importantly, genome-editing approaches that avoid stable integration of foreign DNA may face fewer regulatory constraints in certain jurisdictions, potentially enhancing public acceptance.

Collectively, genetic engineering and genome-editing technologies provide powerful and versatile tools for accelerating biofortification in both staple and horticultural crops. Their application in fruit crops remains an emerging but promising area, with significant potential to complement conventional and agronomic approaches. Representative examples of gene-based and CRISPR-mediated biofortification strategies in fruit crops, including targeted modifications of carotenoid and flavonoid biosynthesis pathways, are summarized in **Table 4**.

**Table 4:** Genetic engineering and CRISPR-based biofortification in fruit crops.

Fruit crop	Gene/target	Source/editing strategy	Biological function	Target trait (nutritional outcome)	Approach	Reference
Apple	Stilbene synthase (STS)	Grapevine gene	Catalyzes resveratrol biosynthesis	Enhanced antioxidant content (resveratrol enrichment)	Transgenic	Flachowsky et al., 2010
Banana	Phytoene synthase (PSY2a)	Maize	Key enzyme in carotenoid biosynthesis	Increased β-carotene (provitamin A)	Transgenic	Waltz, 2014; Paul et al., 2017
Tomato	Lycopene pathway genes (e.g., LCY-E, LCY-B)	CRISPR/Cas9 knockdown	Controls conversion of lycopene to other carotenoids	Increased lycopene accumulation (up to ~5-fold)	CRISPR	Li et al., 2018.
Banana	PDS (Phytoene desaturase)	CRISPR/Cas9 editing	Carotenoid biosynthesis pathway validation	Proof-of-concept for carotenoid biofortification	CRISPR	Kaur et al., 2020
Grapevine	VvWRKY/stilbene pathway genes	CRISPR/Cas9	Secondary metabolite regulation	Increased polyphenols and antioxidant compounds	CRISPR	Ren et al., 2016

### 3.4 Comparative evaluation of biofortification strategies in fruit crops:

The biofortification strategies- agronomic, conventional breeding and genetic approach differs in mechanism, scalability, cost and translational potential. Neither of the single approaches is universally superior (Garg et al., 2018; White and Broadley, 2009). Agronomic biofortification offers the most flexible pathway for nutrient enhancement through foliar or soil application without cultivar replacement or regulatory approval as in the case of conventional breeding (Cakmak et al., 2010). It is highly scalable with low regulatory barriers and generally enjoys high consumer acceptance, though its postharvest stability is variable. However, enhancements are non-heritable and require repeated application of nutrients in comparison to conventional breeding and genetic approaches. Conventional breeding, by contrast is a slow process (often requiring decades) yet it offers heritable, stable improvements with minimal regulatory barriers with an added advantage of stable nutrient retention after harvest. However, it is severely constrained by the long breeding cycles inherent to perennial fruit crops, the limited genetic variation available for many micronutrient traits and the difficulty of decoupling nutritional enhancement from undesirable agronomic trade-offs. Genetic engineering and genome editing occupies a middle ground in terms of speed of implementation and overcome species barriers constraining conventional breeding, enabling precise manipulation of biosynthetic and transport pathways. However, public acceptance and the absence of commercial deployment remain a significant barrier to the widespread adoption of genetically biofortified fruit crops (Saltzman et al., 2013; Garg et al., 2018).

Crucially, no single approach is sufficient to address the different challenges of biofortification. Each strategy targets different aspects of nutrient enhancement and their integration is increasingly recognized as the most promising pathway to achieve sustainable nutritional gains (Cakmak et al., 2010; Bouis and Saltzman, 2017; Bhardwaj et al., 2022). Although these approaches differ in methodology, but their ultimate objective remains the same; improving micronutrient uptake efficiency, transport, storage and accumulation in edible tissues. Agronomic approaches primarily enhance external nutrient availability whereas breeding and genetic engineering modify intrinsic genetic and physiological mechanisms controlling nutrient acquisition and partitioning.

## 4 Biofortification in fruit crops:

Biofortification efforts in fruit crops have increasingly focused on enhancing key micronutrients through crop-specific strategies, including agronomic interventions, conventional breeding, and genetic engineering.

### 4.1 Apple (Malus domestica)

Apple is widely recognized for its rich phytochemical composition, particularly its antioxidant and anti-inflammatory properties, which contribute to its nutritional and functional value (Strand et al., 2018). Given its global consumption, apple represents a promising candidate for micronutrient biofortification, particularly for elements such as iodine and selenium that are often deficient in human diets.

Agronomic biofortification through foliar application has demonstrated significant potential in enhancing micronutrient content in apples. For instance, foliar application of potassium iodide (KI) at 1.0 and 1.5 kg ha^−^¹ has been shown to increase iodine concentrations to approximately 50–100 µg per 100 g fresh weight, which is sufficient to contribute substantially toward the recommended daily intake for adults (Wójcik and Wójcik, 2021). Similarly, selenium enrichment strategies have resulted in a 10–14-fold increase in fruit selenium content (Groth et al., 2020). These findings highlight the effectiveness of agronomic approaches for rapid nutritional enhancement. In contrast, genetic engineering strategies improve functional traits such as the introduction of the stilbene synthase gene from grapevine has led to enhanced resveratrol accumulation, thereby increasing antioxidant capacity (Tian et al., 2017). Furthermore, the development of Arctic^®^ apples through suppression of polyphenol oxidase (PPO) activity has improved post-harvest quality by reducing enzymatic browning, with commercial cultivars such as Fuji, Granny Smith, and Golden Delicious already available (Lobato-Gómez et al., 2021).

### 4.2 Banana (*Musa* spp.)

Banana is a staple fruit crop cultivated extensively across tropical and subtropical regions, many of which are disproportionately affected by micronutrient deficiencies, particularly vitamin A deficiency. Certain banana genotypes naturally accumulate elevated levels of provitamin A carotenoids (pVACs), making them an effective dietary vehicle for delivering essential micronutrients (Amah et al., 2019). To further enhance provitamin A content, genetic engineering approaches have been employed, leading to the development of “Golden Bananas” enriched with β-carotene (Jha et al., 2025). These biofortified varieties aim to address vitamin A deficiency in regions where bananas constitute a major component of the daily diet. Compared to conventional breeding, transgenic approaches enable more targeted enhancement of carotenoid biosynthesis pathways, resulting in significantly higher nutrient accumulation. Agronomic strategies have also been explored to improve mineral nutrition in banana. For example, zinc enrichment through bunch spraying with ZnCl_2_ and bunch stalk feeding with ZnSO_4_ has shown potential in increasing zinc concentration in edible tissues (De Assis et al., 2020). In addition, certain cultivars such as Red Dacca and Red Cavendish exhibit naturally higher levels of vitamin C, potassium, dietary fiber, and anthocyanins, suggesting that conventional varietal selection can complement biofortification efforts (Lobo et al., 2010).

### 4.3 Mango (Mangifera indica)

Mango is a nutritionally important tropical fruit with significant potential for biofortification; however, compared to other fruit crops, targeted biofortification research in mango remains relatively limited. Existing studies have primarily focused on agronomic interventions and preliminary biotechnological approaches, highlighting both opportunities and research gaps.

Agronomic biofortification through foliar application of zinc in different formulations, including zinc oxide nanoparticles (ZnO NPs), zinc sulfate (ZnSO_4_), and chelated zinc, has been reported to significantly improve zinc accumulation in mango fruits (Makhasha et al., 2024). Similarly, combined foliar application of Ca(NO_3_)_2_, H_3_BO_3_, and ZnSO_4_ has demonstrated improvements not only in micronutrient content but also in fruit yield and quality attributes in ‘Dashehari’ mango (Dhotra et al., 2021). These findings suggest that integrated nutrient management strategies can simultaneously enhance productivity and nutritional quality, although their long-term sustainability and nutrient retention efficiency require further evaluation.

In contrast, biotechnological approaches in mango biofortification remain at an early stage. Genetic engineering efforts aimed at enhancing β-carotene content through manipulation of carotenoid biosynthetic pathways have been reported (Saranya et al., 2017). However, these studies are largely experimental and lack field-level validation, limiting their translational applicability.

### 4.4 Pomegranate (*Punica granatum*)

Pomegranate represents a promising candidate for biofortification due to its naturally high content of bioactive compounds and adaptability to diverse agro-climatic conditions. Unlike mango, substantial progress has been achieved in pomegranate through both conventional breeding and agronomic interventions, making it a comparatively more advanced model among fruit crops.

A notable example is the development of the biofortified cultivar ‘Solapur Lal’ by ICAR–National Research Centre on Pomegranate, India. This variety exhibits significantly enhanced micronutrient content, including iron (5.6–6.1 mg/100 g), zinc (0.64–0.69 mg/100 g), and vitamin C (19.4–19.8 mg/100 g), compared to conventional cultivars such as ‘Ganesh’ (Yadava et al., 2020). In addition to its improved nutritional profile, ‘Solapur Lal’ demonstrates high yield potential (23–27 t ha^−^¹) and adaptability to semi-arid regions, highlighting the success of conventional breeding as a sustainable biofortification strategy.

Agronomic biofortification further complements these genetic improvements. Studies have shown that both conventional and nano-formulations of micronutrients significantly enhance fruit quality attributes, including total soluble solids, anthocyanin content, flavonoids, total phenols, and antioxidant activity (Hussein et al., 2024). Notably, nano-micronutrient applications have demonstrated superior efficiency compared to conventional fertilizers, likely due to improved uptake and translocation within plant tissues. Similarly, foliar application of iron sources such as FeSO_4_ and Fe(III)-EDDHA has been reported to positively influence both yield and fruit quality (Davarpanah et al., 2020). These findings underscore the effectiveness of targeted nutrient delivery systems in enhancing both the nutritional and functional properties of pomegranate fruits.

### 4.5 Strawberry (*Fragaria ananassa*)

Strawberry is a high-value horticultural crop recognized for its rich composition of vitamins, antioxidants, and polyphenolic compounds, particularly anthocyanins and ellagitannins, which contribute to its strong nutraceutical profile (Giampieri et al., 2012). These attributes make the strawberry a promising candidate for biofortification aimed at enhancing both micronutrient content and functional quality.

Agronomic biofortification has demonstrated considerable success in strawberry, particularly under controlled cultivation systems. Selenium (Se) enrichment of hydroponically grown strawberry (*Fragaria × ananassa* cv. Elsanta) using sodium selenate (Na_2_SeO_4_) at concentrations of 10 µM and 100 µM resulted in significant increases in fruit selenium content. Notably, a 150 g serving of strawberries treated with 10 µM Se was reported to provide approximately 60 µg of selenium, effectively meeting the lower recommended daily intake (Mimmo et al., 2017). These findings highlight the high efficiency of hydroponic systems for targeted micronutrient delivery, particularly for elements like selenium that are highly responsive to nutrient solution management.

Comparatively, micronutrient accumulation through soil-based fertilization appears less efficient. For instance, iron enrichment in strawberry fruits has been reported to be significantly lower under soil application compared to foliar or aerial delivery methods, due to limited translocation and soil–nutrient interactions (Budke et al., 2020). The foliar application of Na_2_SeO_3_ increased the Se concnetration in strawberries (Lin et al., 2024). This underscores the importance of application method optimization in achieving effective biofortification outcomes.

Genetic engineering strategies aimed at enhancing vitamin C content have shown promise, with concurrent improvements in plant resilience to biotic and abiotic stresses (Parmar et al., 2017). Such dual benefits highlight the synergistic potential of metabolic engineering, where nutritional enhancement is coupled with improved stress tolerance.

### 4.6 Papaya (*Carica papaya*)

Papaya is a nutritionally important tropical fruit, widely recognized for its high content of provitamin A carotenoids, making it a valuable dietary component in regions affected by vitamin A deficiency. Unlike crops such as pomegranate, where direct biofortification strategies have been extensively developed, research in papaya has largely focused on enhancing nutrient bioavailability rather than direct nutrient enrichment. Studies indicate that iron and zinc supplementation can significantly improve the bioavailability and metabolic utilization of provitamin A carotenoids from papaya, particularly in populations consuming vitamin A-deficient diets (Kana-Sop et al., 2015). This highlights an important but often overlooked dimension of biofortification improving nutrient absorption alongside increasing nutrient content.

In contrast to biotechnological perspective, genetic modification approaches have been explored to enhance carotenoid biosynthesis in papaya through the introduction or upregulation of genes involved in the carotenoid pathway (Shankar et al., 2010). However, similar to mango, these approaches remain largely at the experimental stage, with limited evidence of commercial deployment or field-scale validation.

## 5 Bioavailability and human nutritional impact of biofortified fruits

While biofortification strategies aim to enhance the micronutrient concentrations of fruit crops however, enhanced micronutrient accumulation at harvest does not necessarily guarantee nutritional delivery to consumers because nutrient stability during storage, transport and processing critically influence final dietary intake. Thus, the ultimate contribution to human health depends not only on nutrient content but, critically, on bioavailability—the fraction of ingested nutrients that is effectively digested, absorbed, and metabolically utilized by the human body. This distinction is fundamental because increases in nutrient concentration within edible tissues do not necessarily translate into proportional improvements in nutritional status. Moreover, nutrient stability may be compromised during postharvest storage, processing, and culinary preparation, thereby reducing the fraction of nutrients ultimately available for physiological utilization (EFSA Panel; Chungchunlam and Moughan, 2023).

Bioavailability is governed by a complex interplay of factors, including food composition, nutrient–nutrient interactions, the presence of antinutritional compounds, physicochemical characteristics of the food matrix, processing conditions, and host-specific physiological factors (Ofori et al., 2022). In plant-derived foods, antinutrients such as phytic acid, polyphenols (e.g., tannins), and certain dietary fibers can chelate essential minerals, particularly iron and zinc, thereby limiting intestinal absorption. Diets high in phytate are consistently associated with reduced mineral bioavailability, whereas processing interventions such as fermentation, soaking, and enzymatic degradation can substantially enhance nutrient accessibility and absorption efficiency. Thus, the effectiveness of biofortification depends not only on successful nutrient accumulation in crops or their retention during postharvest handling but also on nutrient bioavailability and physiological utilization in humans.

Nutrient interactions further modulate micronutrient utilization. Vitamin C, for example, plays a critical role in enhancing non-heme iron absorption by reducing ferric iron (Fe^3+^) to the more soluble ferrous form (Fe^2+^) and by forming absorbable iron–ascorbate complexes within the intestinal lumen. Conversely, elevated concentrations of calcium and other divalent cations may competitively inhibit the absorption of iron and zinc. In addition, the structural properties of the food matrix strongly influence nutrient release and accessibility during digestion, while the presence of dietary lipids facilitates the absorption of fat-soluble compounds such as provitamin A carotenoids—an important consideration for fruit crops including mango, papaya, and banana (Bouis et al., 2019).

Evidence from *in vitro* digestion assays, stable isotope studies, and human intervention trials indicates that increased micronutrient density in biofortified crops can improve biomarkers of nutritional status, including serum ferritin, plasma retinol, and zinc concentrations (Huey et al., 2024; Gulyas et al., 2025). In this context, biofortification aims not only to increase nutrient concentration but also to improve nutrient bio accessibility and metabolic utilization during digestion and absorption (Malik and Maqbool, 2020; Kumar et al., 2019). Consequently, substantial efforts have focused on developing crops enriched with bioavailable forms of essential micronutrients (Kiran et al., 2022; Umar et al., 2019).

Among fruit crops, banana represents one of the most extensively investigated examples of nutritionally targeted biofortification. Research conducted by Queensland University of Technology, supported by the Bill & Melinda Gates Foundation in collaboration with the National Agricultural Research Organisation in Uganda, led to the development of the biofortified “Super Banana” aimed at addressing vitamin A and iron deficiencies in populations dependent on banana-based diets (Bechoff and Dhuique‐Mayer, 2017). Similarly, genetically modified East African Highland Bananas (EAHBs) enriched with provitamin A carotenoids demonstrated improved nutritional stability under customary cooking conditions. Whereas prolonged cooking of traditional “Matooke” bananas substantially reduces carotenoid content, biofortified lines retained β-carotene equivalent concentrations above the target threshold (21.7–28.7 μg g^−^¹ DW) even after extended cooking periods, thereby supporting their potential nutritional efficacy under real household preparation practices (Buah et al., 2025). These findings are particularly important because they demonstrate that nutrient retention during processing may be as critical as nutrient concentration at harvest.

Comparable observations have been reported in papaya, where provitamin A carotenoids undergo efficient *in vivo* bioconversion to retinol, while concurrent iron and zinc supplementation significantly enhances carotenoid utilization and metabolic efficiency (Kana-Sop et al., 2015). Furthermore, the naturally high vitamin C content of many fruits provides an additional nutritional advantage by enhancing non-heme iron absorption, thereby potentially improving the efficacy of iron biofortification strategies.

Despite these promising findings, important limitations remain. Most clinical validation studies investigating biofortification efficacy have focused primarily on staple crops such as pearl millet, rice, maize, and wheat, whereas direct human evidence for biofortified fruit crops remains comparatively limited. This represents a major research gap that must be explicitly acknowledged. Although it is plausible that biofortified fruits may confer nutritional benefits analogous to those reported for staple crops, direct extrapolation remains scientifically uncertain due to substantial differences in food matrix composition, dietary consumption patterns, culinary processing methods, and micronutrient chemical forms between fruits and cereals.

Moreover, the literature reveals important inconsistencies between experimental systems. While *in vitro* bio accessibility assays frequently report high mineral recovery from fruit matrices, actual *in vivo* absorption may be considerably lower because of competitive nutrient interactions, the presence of tannins and phytates in certain fruits, and interindividual physiological variability (Ofori et al., 2022). Similar uncertainties exist for provitamin A carotenoids, where conversion efficiency to retinol is highly variable and strongly influenced by dietary composition, lipid availability, nutritional status, and host metabolism. Reported retinol equivalence ratios vary widely across studies, indicating that carotenoid concentration alone cannot reliably predict vitamin A nutritional impact (Saltzman et al., 2013). Collectively, these inconsistencies highlight the urgent need for standardized, fruit-specific bioavailability protocols and rigorously controlled human intervention studies before definitive nutritional efficacy claims can be established for biofortified fruit crops.

Importantly, fruits possess several intrinsic advantages over staple crops in the context of micronutrient delivery. Unlike cereals and legumes, fruits are naturally rich in compounds such as vitamin C, organic acids, and carotenoids that may facilitate improved micronutrient absorption. However, these advantages are counterbalanced by challenges associated with high moisture content, perishability, seasonal availability, and variability in consumption frequency, all of which may constrain consistent nutrient delivery at the population level. Consequently, the nutritional value of biofortified fruits must be evaluated not only in terms of nutrient enrichment but also in relation to dietary context, postharvest stability, processing behavior, and long-term consumption patterns.

Another critical consideration is that micronutrient enhancement must achieve biologically meaningful thresholds to produce measurable improvements in human health. Marginal increases in nutrient concentration may be insufficient to improve nutritional outcomes, particularly in populations affected by multiple micronutrient deficiencies, chronic malnutrition, or low dietary diversity.

In addition, several interconnected constraints continue to limit the translation of crop-level biofortification into tangible public health outcomes. These include genotype × environment interactions affecting nutrient stability, climate change-induced nutrient dilution effects, postharvest nutrient degradation, limited understanding of nutrient interactions within complex diets, insufficient clinical validation, weak integration with public health frameworks, and economic as well as regulatory barriers that constrain large-scale implementation.

Advancing fruit crop biofortification therefore requires a systems-oriented and interdisciplinary framework integrating plant breeding, agronomic optimization, postharvest biology, food science, and human nutrition research. Strengthening the evidence base through biomarker-driven intervention trials, rigorous bioavailability assessments, and long-term nutritional evaluation will be essential to substantiate the role of biofortified fruits in combating micronutrient malnutrition.

Overall, current evidence supports the potential of biofortified fruits as an important component of nutrition-sensitive agriculture. However, their long-term contribution to global nutritional security will ultimately depend on successfully bridging the gap between enhanced nutrient concentration in crops and demonstrable improvements in human health outcomes.

## 6 Research gaps and limitations

Despite notable advances in fruit crop biofortification, several interrelated constraints continue to limit its scalability and translation into measurable public health outcomes.

A primary limitation lies in the disconnect between nutrient concentration and nutritional efficacy. While many studies report enhanced micronutrient levels in fruit tissues, these increases do not necessarily translate into improved bioavailability or significant changes in human nutritional status. The lack of robust human intervention trials evaluating long-term health outcomes of biofortified fruit consumption remains a critical evidence gap, hindering policy integration and large-scale implementation. At the crop level, genotype × environment (G × E) interactions play a decisive role in determining micronutrient accumulation. Variability in soil composition, climatic conditions, and agronomic practices can significantly influence nutrient content, yet the stability of biofortified traits across diverse agro-ecological regions is insufficiently characterized. This challenge is further exacerbated by climate change, which alters soil nutrient dynamics, temperature regimes, and water availability, potentially leading to nutrient dilution and reduced effectiveness of biofortification strategies.

Postharvest factors represent another underexplored constraint. Nutrient degradation during storage, transportation, and processing can substantially reduce the micronutrient content available to consumers, thereby limiting real-world nutritional impact. Despite its importance, postharvest nutrient retention in biofortified fruits remains inadequately studied, particularly under supply chain conditions typical of developing regions. Biological and genetic complexities inherent to fruit crops also pose significant challenges. Perennial growth habits, long juvenile phases, polyploidy, and complex genomes slow breeding progress and complicate trait integration. In addition, the limited functional characterization of genes involved in micronutrient uptake, transport, and accumulation restricts the efficiency of both conventional breeding and genome-editing approaches. Beyond biological constraints, socio-economic and regulatory barriers significantly influence adoption. Regulatory uncertainties surrounding genetically engineered and genome-edited crops, coupled with biosafety concerns and variable consumer acceptance, can delay deployment. Furthermore, the economic feasibility of agronomic biofortification, particularly the cost of micronutrient-enriched fertilizers remains a challenge for smallholder farmers. Issues related to seed and planting material distribution, accessibility, and cost-benefit trade-offs are often overlooked in current research frameworks.

Another critical gap is the predominant focus on single-nutrient biofortification, despite the widespread coexistence of multiple micronutrient deficiencies in vulnerable populations. This highlights the need for integrated, multi-nutrient biofortification strategies that better reflect real-world dietary deficiencies.

**Figure 3:**
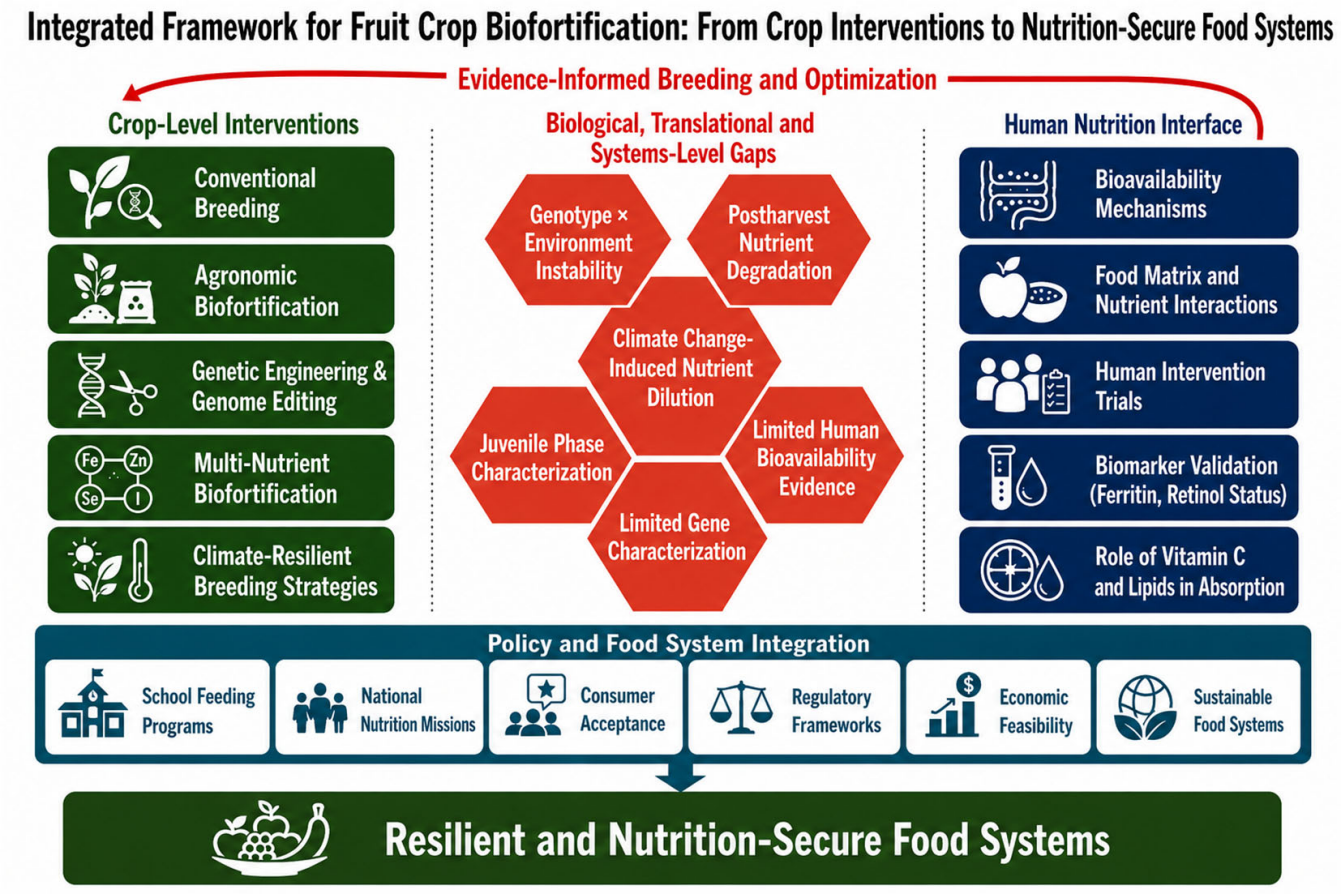
Conceptual framework linking biofortification strategies in fruit crops to human nutritional outcomes. The framework illustrates the pathway from crop-level interventions (genetic, agronomic, and biotechnological approaches) to improved micronutrient intake and nutritional status. Key constraints include genotype × environment interactions, climate-driven nutrient variability, postharvest losses, and limited evidence on bioavailability. The role of nutrient interactions, bioavailability, and policy integration in achieving nutrition-sensitive and sustainable food systems is also highlighted.

Addressing these challenges requires a systems-based, interdisciplinary approach that integrates plant science, human nutrition, socio-economic analysis, climate adaptation, and policy frameworks. Future research should prioritize long-term field validation, standardized methodologies for assessing nutritional impact, and alignment with national and global nutrition strategies. Strengthening these areas will be essential to fully realize the potential of fruit crop biofortification as a sustainable solution to hidden hunger. These interconnected biological, environmental, and socio-economic constraints are conceptually synthesized in a systems-level framework (**Figure 3**).

## 7 Conclusion

**Figure 4:**
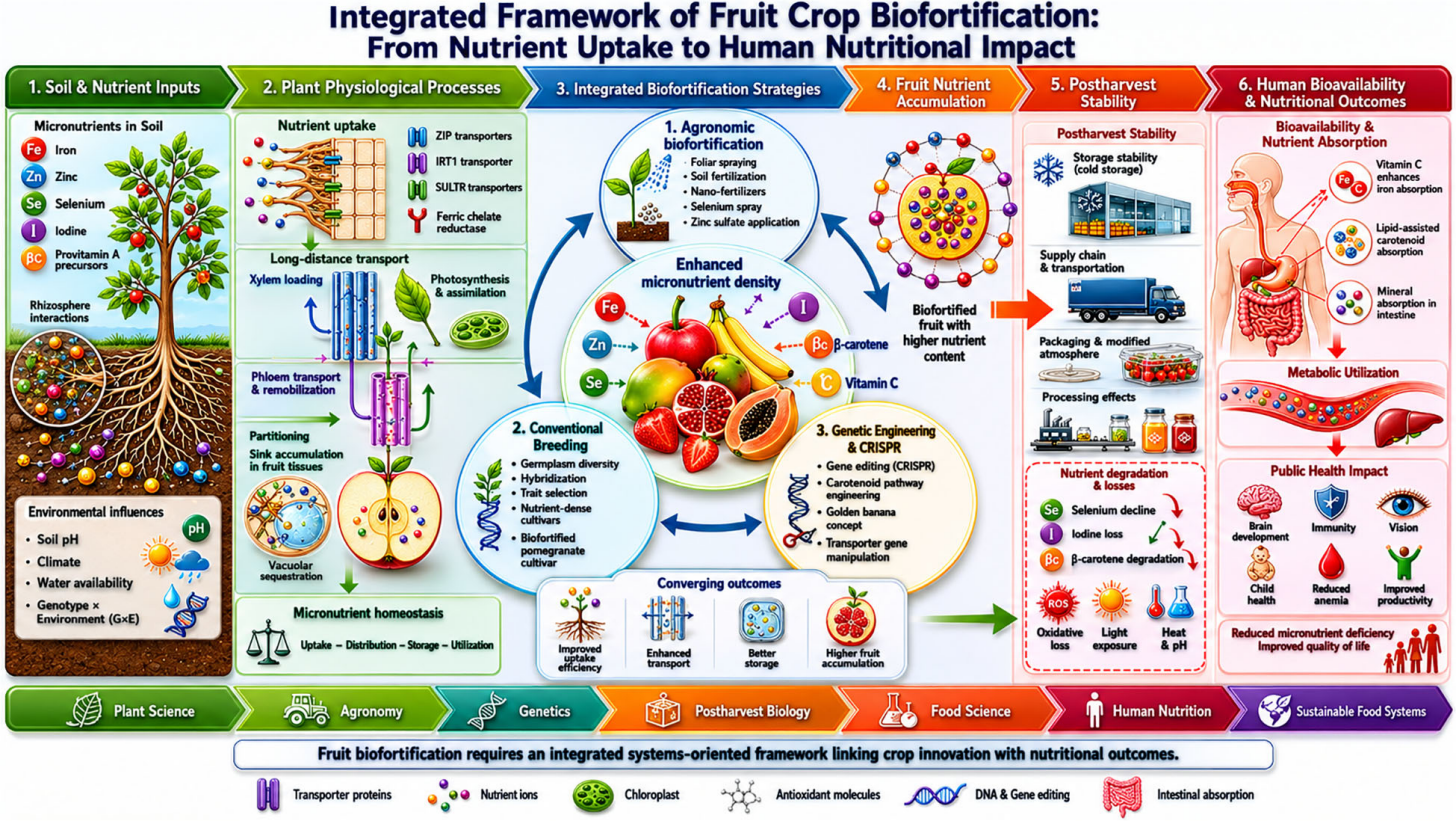
A conceptual summary figure integrating physiological mechanisms of nutrient uptake and transport, biofortification strategies, postharvest nutrient stability, and human nutritional outcomes. The framework illustrates sequentially and mechanistically connected components of a unified biofortification pathway, rather than parallel independent elements.

Fruit crop biofortification represents a promising nutrition-sensitive strategy for addressing persistent micronutrient deficiencies, particularly in regions where hidden hunger persists despite gains in food production. While agricultural intensification has substantially improved yield and caloric availability, it has not consistently enhanced dietary quality, highlighting the need to integrate nutritional traits into crop improvement frameworks. In this context, fruit crops owing to their widespread consumption, cultural acceptability, and inherent richness in vitamins, minerals, and bioactive compounds offer significant potential as effective vehicles for micronutrient delivery. However, increasing nutrient concentrations in edible tissues alone is insufficient to achieve meaningful public health outcomes. Translating crop-level nutritional enhancement into human health benefits requires careful consideration of bioavailability, postharvest nutrient stability, and dietary context, alongside robust clinical validation through well-designed human intervention studies. Furthermore, genotype × environment interactions and climate-driven shifts in soil and plant nutrient dynamics may significantly influence micronutrient accumulation, necessitating adaptive breeding strategies and location-specific agronomic interventions. Future progress in fruit biofortification will depend on the strategic integration of conventional breeding, genome editing technologies, and precision agronomic practices to develop nutritionally enhanced, stable, and high-performing cultivars. A shift toward multi-nutrient biofortification is particularly critical, given the widespread coexistence of multiple micronutrient deficiencies in vulnerable populations. At the same time, enabling factors such as supportive regulatory frameworks, consumer acceptance, and economic feasibility must be addressed to ensure scalability and equitable access. Importantly, the incorporation of biofortified fruit crops into public health and nutrition programs including school feeding initiatives and community-based food systems offers a viable pathway to amplify their impact. When implemented in synergy with existing interventions such as supplementation and food fortification, fruit biofortification can contribute to building resilient, nutrition-secure, and sustainable food systems. Overall, realizing the full potential of fruit crop biofortification will require a coordinated, multidisciplinary approach that bridges plant science, agronomy, genetics, postharvest biology, food science, and human nutrition toward resilient and nutrition-secure food systems as shown in the **Figure 4**. Strengthening these linkages is essential to transform biofortified fruits from a promising innovation into a scalable and impactful solution for global nutrition security.
